# Supplementary figures and images for: Effects of Wnt10a and Wnt10b Double Mutations on Tooth Development
Source: Genes (Basel). 2023 Jan 28;14(2):340. doi: 10.3390/genes14020340 (PMC9957325; doi:10.3390/genes14020340)

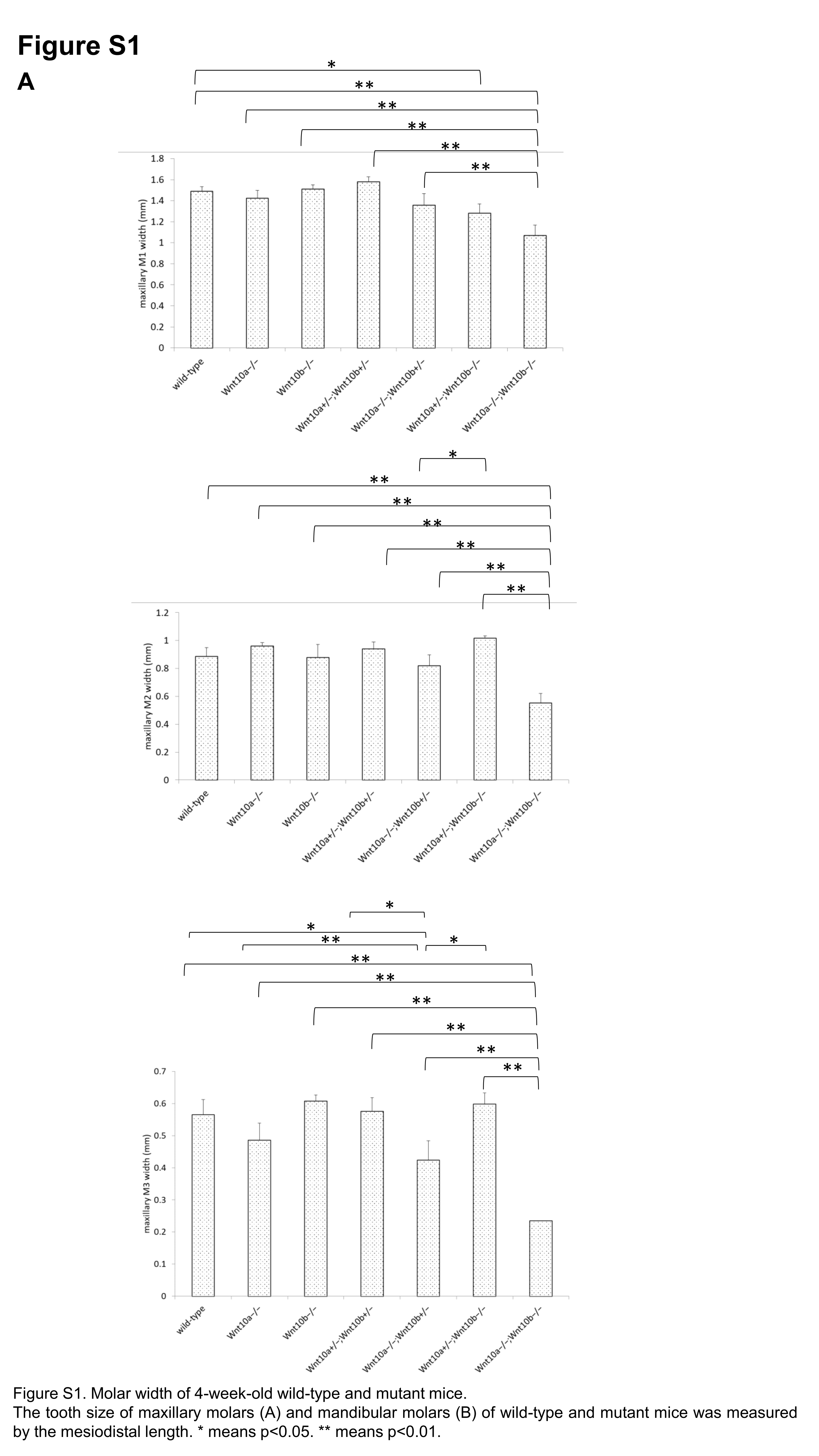

Supplement: Supplementary file 1 [file genes-14-00340-s001.zip › Figure S1A.20230127.tif]

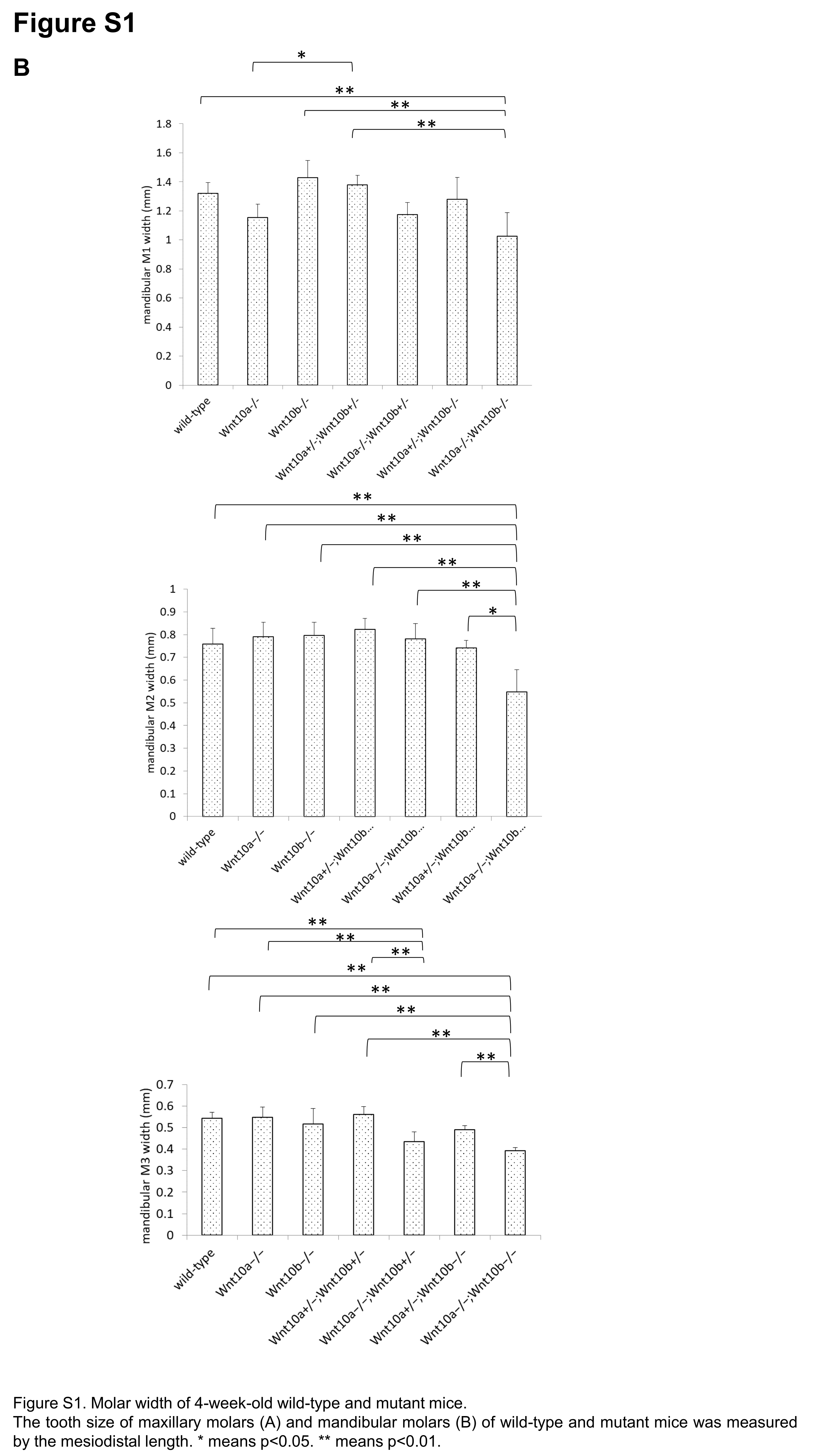

Supplement: Supplementary file 1 [file genes-14-00340-s001.zip › Figure S1B20230127.tif]
